# 

**Published:** 1830-04-01

**Authors:** 


### CONTENTS

OF THE

### MEDICO-CHIRURGICAL REVIEW.

No. XXIV. APRIL 1, 1830.

### REVIEWS.

I.

Popular Illustrations of Medicine. By SHIRLEY PALMER, M.D. .. .. .. .. 289

II.

A Practical Treatise on Acute Abdominal and Pelvic Inflammation. By Mr. BATES 313

III.

Dr. PRICHARD on Physical and Animal Life.. .. .. .. .. .. .. .. . 316

IV.

Dr. GOOCH on Polypus of the Uterus .. .. .. .. .. .. .. .. .. .. .. 321

V.

Dr. CORDEN THOMPSON on Anatomical Pursuits .. .. .. .. .. .. .. .. .. 332

VI.

Dr. ARNOTT's Elements of Physics .. .. .. .. .. .. .. .. .. .. .. .. .. 334

VII.

FEVER.

1. A Treatise on Fever. By SOUTHWOOD SMITH, M.D. .. .. .. .. .. .. } 337

2. Clinical Illustrations of Fever. By ALEXANDER TWEEDIE, M.D... .. .. }

VIII.

Principles of Military Surgery. By JOHN HENNEN, MD... .. .. .. .. .. .. 361

IX.

A Treatise on Poisons. By ROBERT CHRISTISON, M.D .. .. .. .. .. .. .. 362

X.

Pathological Researches on Inflammation of the Veins of the Uterus. By ROBERT LEE, M.D .. .. .. .. .. .. .. .. .. .. .. .. .. .. .. .. .. 376

XI.

Drs. SMITH and TWEEDIE on Fever—[Concluding Article] .. .. .. .. .. 385

XII.

Consolations in Travel, or the last Days of a Philosopher. By Sir H. DAVY, Bart... 401

XIII.

Practical Remarks on Amputations, Fractures, and Strictures of the Urethra. By Mr. HAMMICK .. .. .. .. .. .. .. .. .. .. .. .. .. .. .. .. 405

XIV.

Dr. SEYMOUR on Ovarian Dropsy .. .. .. .. .. .. .. .. .. .. .. .. .. 416

### PERISCOPE.

1. Lunacy *versus* Sanity—Law *versus* Physic .. 433
2. Employment of the Suture in Vesico-vaginal Fistula .. 437
3. Liberty of the Press—Club-law .. 438
4. Cases of Extirpation of the Ovaries .. 440
5. Spontaneous Purification of Thames Water .. 443
6. Unrecognized Cystocele .. 444
7. Curriculum of the Irish College of Surgeons, with Strictures .. 446
8. On the Diagnosis of Hernia .. 450
9. Opinions of Foreigners respecting English Medical Politics .. 454
10. Interesting Cases of Apoplexy .. 457
11. Seat of the Soul! .. 461
12. Dr. England on Disorders of the Kidneys .. 481
13. Mr. Cameron on Nitre in Scurvy .. 483
14. Provident Medical Institution of Scotland .. 484
15. Remuneration of General Practitioners .. 486
16. A. Chronic Mollescence of the Spinal Cord .. 487
B. Caries of the Odontoid Process .. 489
17. Recamier on Cancer .. 490
18. Action of Air and Water on Lead .. 491
19. Morbid Itching of the Scrotum .. 493
20. Anatomical Description of a Chinese Foot .. 494
21. Tests for Oxalic Acid .. 496
22. Anomalous Affection of the Scrotum .. 498
23. Mr. Marshall on Vaccination .. 499
24. Mr. Scott on Lavements .. 499
25. Operation for Carotid Aneurism .. 501
26. Epilepsy cured by Trephining .. 504
27. Fatal Stricture of the Colon .. 504
28. Elephantiasis of the Scrotum and Labia Pudendi .. 505
29. Hypochondriasis .. 509
30. Sane or Insane .. 509
31. Injurious Effects of Tight Lacing .. 512
32. Mr. Jewel on Nitrate of Silver in Leucorrhœa .. 517
33. Dinner of the General Practitioners .. 518
34. College of Physicians (first Meeting) .. 520
35. On Dissection-Wounds. By Wm. Lawrence, Esq. with Remarks .. 529
36. Intermittent Ophthalmia .. 534
37. Enormous Swelling of the Scrotum, resembling Elephantiasis, with the Operation. By M. Delpech .. 535
38. M. Dupuytren's Treatment of Specks on the Cornea .. 539
39. Dr. Barry on the Gibraltar Fever .. 539
40. A Practical Formulary of the Parisian Hospitals. By Dr. M'Lellan .. 540
41. M. Bally on the Therapeutic Effects of Morphine .. 545
42. A Treatise on Syphilis. By John Bacot, Esq. .. 548
43. Mr. M'Fadzen on Water-dressing in Wounds, Ulcers, Diseases of the Skin, &c. 551
44. On the Diseases of Children. By Mr. Marley .. 553
45. Dupuytren's Treatment of Scrofula .. 554

### CLINICAL REVIEW.

### St. George's Hospital.

1. Umbilical Hernia—Dropsy—Asthma—Enormous Hypertrophy of the Heart .. 463
2. Interesting Case of Pneumo-Thorax .. 466
3. On the Connexion between Pulmonary Apoplexy and Contraction of the Left Auriculo-Ventricular Opening of the Heart .. 555
   Case 1 .. 557
   Case 2 .. 558
   Case 3 .. 559
   Case 4 .. 560
   Other Cases .. 561

### Massachusett's General Hospital.

1. Amputation of the Jaw .. 469
2. Excision of Nerves in Cases of Neuralgia .. 470

### Edinburgh Surgical Hospital.

Mr. Syme on the Treatment of Ulcers .. 474

### Hôpital de la Pitie.

Aneurism after Venesection .. 476

### Philadelphia Infirmary.

Cases of obscure Peritonitis .. 478

### St. Louis Hospital.

1. Cæsarean Operation after Death .. 521
2. Severe Symptoms after opening a Body .. 522
3. M. Alibert on Leprous Affections .. 523

### Glasgow Royal Infirmary.

1. Tumours of the Female Breast .. 524
2. On Loose Cartilages in the Knee-joint .. 562
3. Fractures, Simple and Compound .. 566
4. Delirium Tremens .. 568
5. Burns .. 570
6. Lupus treated by the Iodide of Mercury .. 570

### South-Eastern District General Dispensary.

Case of Fungus Hæmatodes of the Stomach and Liver. By Dr. Thwaites .. 563

### Madras Military Hospital.

Mr. Annesley on Elongation and unnatural Positions of the Colon .. 570

### MISCELLANIES.

1. College of Surgeons .. 528
2. Reflections on the Morbid Phenomena caused by the Use of Iodine. By Dr. Jahn 572
3. Carded Cotton to Blistered Surfaces .. 572
4. Anatomy Bill. Mr. Guthrie's Lectures .. 573
5. Hunterian Society .. 574
6. North of England Medical and Surgical Journal .. 574
7. Bibliographical Record .. 575

